# Case Report: Converting an immunologically “cold” tumor: exceptional response to cadonilimab plus chemotherapy in microsatellite-stable pancreatic cystadenocarcinoma

**DOI:** 10.3389/fimmu.2026.1735785

**Published:** 2026-05-29

**Authors:** Chunxiao Ni, Jiaju Xu, Yu Pang, Jiaju Xu

**Affiliations:** 1 Department of Minimally Invasive Oncology, Tai’an City Central Hospital, Tai’an, Shandong, China; 2 Department of Pediatrics, Yantai Yuhuangding Hospital, Yantai, Shandong, China; 3 Department of Pathology, Tai’an City Central Hospital, Tai’an, Shandong, China; 4 Department of Medical Oncology, Tai’an City Central Hospital, Tai’an, Shandong, China

**Keywords:** cadonilimab, bispecific antibody, pancreatic cystadenocarcinoma, microsatellite stable, complete remission, immunotherapy

## Abstract

**Background:**

Pancreatic cancer (PC) remains largely refractory to immune checkpoint inhibitors (ICIs), especially in the prevalent microsatellite-stable (MSS) subtype. However, combination strategies of ICIs with chemotherapy have largely failed in MSS PC, highlighting an urgent need for novel immunotherapeutic approaches. Cadonilimab is a novel programmed death protein 1(PD-1)/cytotoxic T-lymphocyte-associated protein 4 (CTLA-4) bispecific antibody designed to remodel the immunosuppressive tumor microenvironment.

**Case report description:**

We report an exceptional and durable response to cadonilimab plus chemotherapy in a patient with metastatic MSS pancreatic cystadenocarcinoma (PCAC). Microsatellite stability was shown by immunohistochemistry with intact expression of all four mismatch repair proteins (MLH1, MSH2, MSH6, PMS2) and confirmed by next-generation sequencing (NGS). Following disease progression on first-line gemcitabine/nab-paclitaxel and transarterial chemoembolization (TACE), the patient subsequently received second-line therapy with cadonilimab combined with nab-paclitaxel and oxaliplatin. Notably, within two months of treatment initiation, the patient achieved near-complete remission (near-CR) per Response Evaluation Criteria in Solid Tumors (RECIST) 1.1 criteria, with an 89.9% reduction in the sum of target lesion diameters. Despite discontinuing anticancer therapy after 4 cycles due to grade 3 bone marrow suppression, which subsequently evolved into and has been maintained as a complete response (CR) for over 30 months, with the primary endpoint of progression-free survival (PFS) not yet reached.

**Conclusion:**

This case provides pioneering clinical evidence that cadonilimab, in combination with chemotherapy, can induce profound and durable remission in MSS PCAC, challenging current paradigms of ICI resistance and supporting the further development of bispecific antibody strategies.

## Introduction

Pancreatic cancer (PC) remains one of the most lethal malignancies, with a 5-year overall survival rate of 13% (1). Pancreatic cystadenocarcinoma (PCAC), a rare mucin-producing subtype constituting about 1% of all PCs, is historically associated with a modestly more favorable prognosis than pancreatic ductal adenocarcinoma (PDAC) in some retrospective series, though outcomes for advanced disease remain poor (2, 3). However, for patients with advanced or metastatic disease, therapeutic options are severely limited and outcomes remain dismal, with a median survival of just 4 months post-metastasis and no established standard of care beyond regimens borrowed from PDAC (4–6).

The advent of immune checkpoint inhibitors (ICIs) has revolutionized the treatment of many solid tumors, but their efficacy in PC, including PCAC, has been largely confined to the rare microsatellite instability-high (MSI-H) subtype (7, 8). The vast majority of PCs are microsatellite stable (MSS) and characterized by an immunologically “cold” tumor microenvironment (TME)—marked by low tumor mutational burden, low PD-L1 expression, and profound immunosuppression—which renders them refractory to programmed death protein 1(PD-1)/programmed death-Ligand 1 (PD-L1) and cytotoxic T-lymphocyte-associated protein 4 (CTLA-4) monotherapy (9, 10). While combination strategies, such as ICIs with chemotherapy, have been explored to convert this “cold” TME, these efforts have largely failed to yield significant clinical benefit in MSS PC populations (11, 12).

Cadonilimab is a first-in-class bispecific antibody targeting both PD-1 and CTLA-4. This unique design may potentially achieve synergistic immune activation by concurrently blocking distinct inhibitory pathways, offering a promising strategy to overcome the formidable immunosuppressive barriers in MSS tumors (13).

Herein, we report the first case of a patient with metastatic MSS PCAC who achieved a profound and durable remission, culminating in a sustained complete response. following treatment with cadonilimab in combination with chemotherapy. This exceptional response challenges the prevailing paradigm of ICI resistance in MSS pancreatic cancers and provides compelling rationale for the further investigation of bispecific antibody-based immunotherapy in this aggressive disease.

## Case presentation

**Figure 1:**
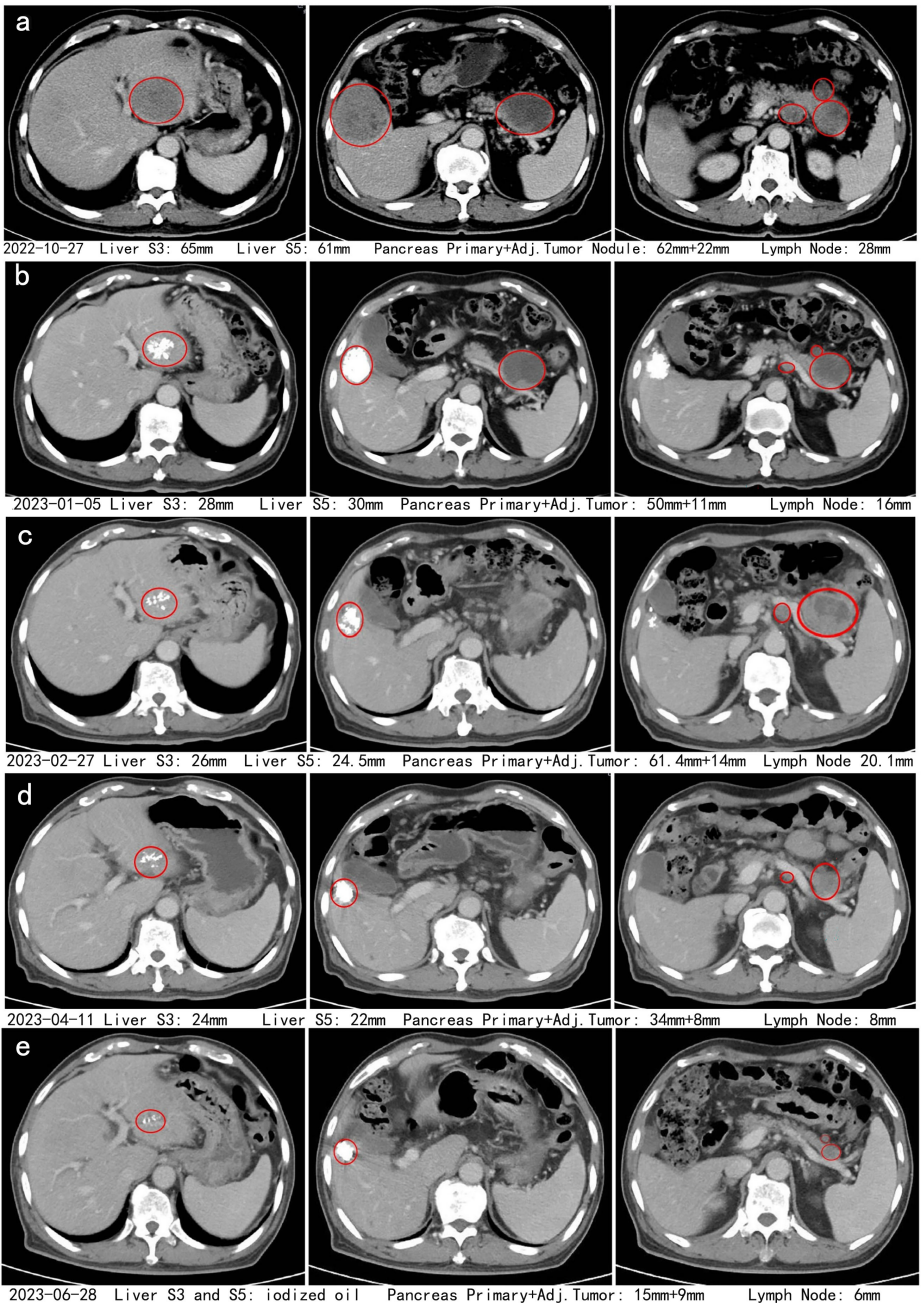
Serial abdominal computed omography (CT) images (axial view) during treatment. Red circles indicate the target lesions. For each of the five key time points (a–e), representative CT images at three consecutive anatomical levels are displayed, with the examination dates annotated. **(a)** Baseline (October 2022): A solid mass is seen in the pancreatic tail (yellow arrow, approximately 62 × 43 mm), with an adjacent cystic-solid tumor nodule located anterosuperiorly (related to the primary tumor, approximately 22 × 18 mm). Liver metastases are present in segments S3 and S5 (approximately 61 × 61 mm and 65 × 60 mm, respectively). Enlarged metastatic lymph nodes are noted inferior to the pancreatic body (approximately 28 × 18 mm). **(b)** Partial Response (January 2023): After first−line therapy (TACE plus gemcitabine/nab−paclitaxel), the primary pancreatic tail lesion, the adjacent cystic-solid tumor nodule, liver metastases (with iodized oil deposits), and metastatic lymph nodes all showed significant reduction in size. **(c)** Progressive Disease (February 2023): The primary pancreatic tail lesion (yellow arrow) and abdominal metastatic lymph nodes increased in size, consistent with disease progression (**Table 1**); the liver metastases (areas of iodized oil deposition) decreased in extent compared with prior imaging. **(d)** Marked Response (April 2023): After two cycles of cadonilimab combination chemotherapy, the primary pancreatic tail lesion, the adjacent cystic lesion, liver metastases (predominantly iodized oil deposits with further reduction in extent), and metastatic lymph nodes all demonstrated marked shrinkage, achieving a partial response (**Table 1**). **(e)** Near−Complete Response (June 2023): Following discontinuation of cadonilimab and completion of two subsequent cycles of chemotherapy, the enhancing lesions in the pancreas and lymph nodes were significantly reduced, with only stable iodized oil deposits remaining in the liver, corresponding to a near−complete response (**Table 1**).

A 68-year-old male presented in October 2022 with a two-month history of dull abdominal pain and discomfort. Cross-sectional imaging revealed a locally advanced pancreatic tail mass, an adjacent cystic-solid tumor nodule, synchronous bilobar liver metastases and extensive lymph node involvement, and the disease was staged as cT4N2M1 (Stage IV) according to the American Joint Committee on Cancer (AJCC) Cancer Staging Manual, 8th edition (**Figure 1a**). The diagnostic workup proceeded in a stepwise manner, beginning with a US-guided biopsy of a liver metastasis, which established the diagnosis of a poorly differentiated adenocarcinoma. The initial immunohistochemistry (IHC) profile was suggestive of a pancreatobiliary origin but not entirely specific. Based on this finding and the clinical presentation, first-line systemic therapy with gemcitabine and nab-paclitaxel, alongside transarterial chemoembolization (TACE) for the liver metastases, was initiated.

**Figure 2:**
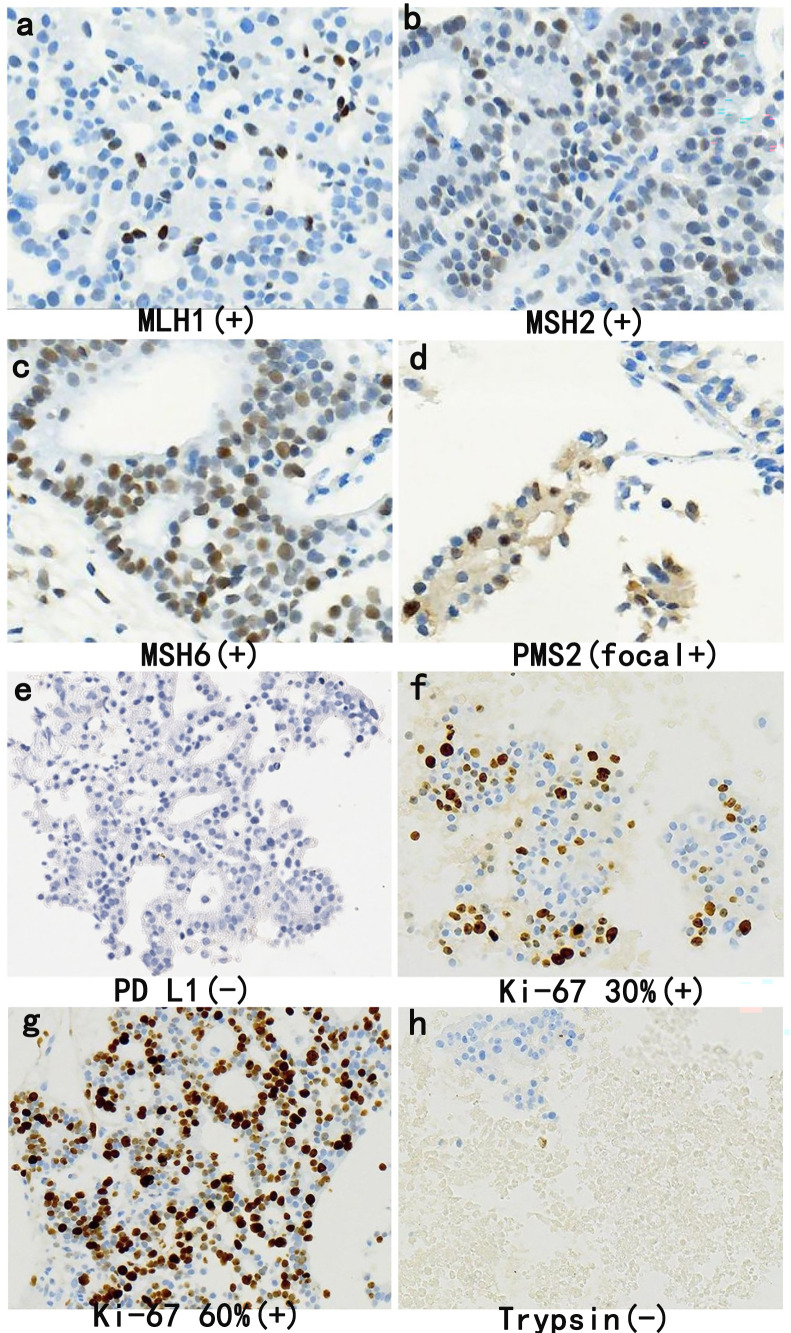
Immunohistochemical (IHC) characterization of mismatch repair status and other markers. **(a-d)** IHC for Mismatch Repair (MMR) proteins performed on the liver metastasis biopsy, confirming proficient MMR (pMMR) status: **(a)** MLH1 (intact nuclear+), **(b)** MSH2 (intact nuclear+), **(c)** MSH6 (intact nuclear+), **(d)** PMS2 (focal but retained nuclear+). **(e-h)** Additional predictive and diagnostic markers: **(e)** PD-L1 (22C3 assay, Combined Positive Score <1, negative) in the liver metastasis. **(f)**Ki-67 (approximately 30%) in the primary pancreatic tumor. **(g)** Ki-67 (approximately 60%) in the liver metastasis. **(h)** Trypsin (negative) in the liver metastasis, aiding in the exclusion of acinar cell carcinoma. All images at ×200 magnification. MMR status was assessed on the diagnostic liver biopsy; the primary tumor specimen was obtained after two cycles of therapy and was not evaluated for MMR proteins.

However, following an initial radiological response that was short-lived, with subsequent disease progression specifically in the pancreatic primary and lymph nodes, the multidisciplinary team (MDT) determined that a more definitive tissue diagnosis was imperative to guide further therapy. Consequently, an endoscopic ultrasound-guided fine needle aspiration (EUS-FNA) of the primary pancreatic tail mass was performed. The cytological specimen revealed clusters of malignant cells with acinar features. The comprehensive IHC profile, now integrating results from both the liver and pancreatic biopsies, was critical for the final diagnosis: the tumor cells were consistently CK7 positive, CK20 negative, and exhibited patchy staining for CDX2 and Villin, while being negative for markers of neuroendocrine (Syn, CgA), hepatocellular (HepPar1), and prostatic (PSA, PSAP) differentiation. Crucially, the tumor cells were also negative for trypsin and chymotrypsin, ruling out acinar cell carcinoma. This integrated pathological evidence ultimately confirmed the diagnosis of PCAC. Furthermore, molecular characterization demonstrated the tumor to be microsatellite stable (MSS/proficient mismatch repair [pMMR]), with intact nuclear expression of MLH1, MSH2, MSH6, and PMS2 (focal+) (**Figure 2**), a finding subsequently confirmed by NGS. PD-L1 expression was negative (Combined Positive Score <1). The proliferative index (Ki-67) was approximately 30% in the primary pancreatic tumor and 60% in the biopsied liver metastasis (**Figure 2**).

The MDT initiated first-line therapy with gemcitabine plus nab-paclitaxel, concurrently with a single session of TACE for the liver metastases. Restaging computed tomography (CT) in January 2023 demonstrated a partial response, as per the Response Evaluation Criteria in Solid Tumors (RECIST) version 1.1, with significant reduction in the primary pancreatic lesion, liver metastases showing dense iodized oil retention, and abdominal lymph nodes (**Figure 1b**). The patient received two additional cycles of gemcitabine and nab-paclitaxel. However, restaging CT on February 27, 2023, revealed an increase in size of the pancreatic primary lesion (from 50.0 to 61.4 mm) and abdominal lymph nodes, with the sum of diameters increasing to 133.0 mm. This clear progression of the primary tumor guided the clinical decision to change therapy (**Tables 1**, **2**, **Figure 1c**).

**Table 1:** Timeline.

2022.10 2022.11	2022.12 2023.01	2023.02 2023.04	2023.05 2023.06	2023.07 2025.10
First-line treatment: one cycle of chemotherapy with abraxane plus gemcitabin and transarterial chemoembolization (TACE)	Two cycles of chemotherapy combining abraxane and gemcitabin	Second-line treatment: two cycles of combination therapy with cadonilimab, abraxane, and oxaliplatin	Two cycles of chemotherapy combining abraxane and oxaliplatin	Owing to the adverse effects experienced from chemotherapy, the patient declined further antitumor therapy, while the disease remained in complete remission (CR)

**Table 2:** Treatment timeline and objective tumor response assessment (RECIST 1.1).

Date (yyyy-mm)	Treatment phase	Target lesion measurements (Pancreas Primary+Adj. Tumor Nodule; Liver S3; Liver S5; Lymph Node; mm)	Sum of diameters (mm)	% Change from baseline	Recist 1.1 response
2022-10-27	Baseline (Diagnosis)	62.0 + 22.0; 61.0; 65.0; 28.0	238.0	--	--
2023-01-05	Post 1st-line (TACE + Gem/Nab)	50.0 + 11.0; 28.0; 30.0; 16.0	135.0	-43.3%	PR
2023-02-27	Progressive Disease	61.4 + 14.0; 26.0; 24.5; 20.1	146.0	-38.7%	PD
2023-04-11	Post 2 cycles (Cadonilimab + Nab/Oxa)	34.0 + 8.0; 24.0; 22.0; 8.0	96.0	-59.7%	PR
2023-06-28	Post 2 cycles (Nab/Oxa)	15.0 + 9.0; (iodized oil); (iodized oil); 0.0	24.0	-89.9%	Near-CR
2024-01-02	Follow-up	0.0; (iodized oil); (iodized oil); 0.0	0.0	-100%	CR
2025-10-15	Follow-up	0.0; (iodized oil); (iodized oil); 0.0	0.0	-100%	CR

TACE, Transarterial chemoembolization; Gem/Nab, Gemcitabine plus nab-paclitaxel; Nab/Oxa, Nab-paclitaxel plus oxaliplatin; PR, Partial Response; PD, Progressive Disease; Near-CR, Near-Complete Response; CR, Complete Response.

Adj. stands for Adjacent, referring to the cystic-solid tumor nodule contiguous with the primary pancreatic mass. The measurement for “Pancreas Primary+ Adj. Tumor Nodule” represents the sum of the longest diameters of the two contiguous tumor sites in the pancreas.

Liver measurements at 2023-01, 2023-02, and 2023-04 primarily reflect iodized oil deposits from prior TACE, not necessarily viable tumor. By 2023-06 and thereafter, only stable, non-enhancing iodized oil deposits were visualized in the liver, with no evidence of enhancing tumor components.

Complete Response (CR), disappearance of all target lesions; Partial Response (PR), ≥30% decrease in sum; Progressive Disease (PD), ≥20% increase in sum and ≥5 mm absolute increase, or unequivocal progression of non-target lesions. PD in Feb 2023 was based on the clear growth of the pancreatic primary lesion.

The assessment of “Progressive Disease (PD)” in February 2023 was primarily driven by a clear increase in size of the pancreatic primary lesion and lymph node, which guided the clinical decision to change therapy, consistent with clinical progression despite a less than 20% increase in the total sum of diameters.

The Sum of Diameters and % Change for 2023-06 are calculated based on enhancing lesions only (Pancreas, 15.0 mm; Adj. Tumor Nodule, 9.0 mm), as the liver findings represented inert material.

Following MDT re-evaluation, the treatment strategy was adjusted in light of the disease progression on first-line therapy. The MDT noted that the cystic nature of the metastases suggested limited benefit from anti-angiogenic agents, and the therapeutic effect on liver lesions was primarily attributed to prior TACE. Given the elevated AFP levels and the lack of efficacy of gemcitabine plus nab-paclitaxel, the decision was made to initiate a second-line regimen combining immune checkpoint inhibition with a different chemotherapy backbone. Although the patient declined participation in clinical trials at this time, and PD-L1 testing returned negative, the regimen of cadonilimab (a PD-1/CTLA-4 bispecific antibody) plus nab-paclitaxel and oxaliplatin was instituted. A follow-up CT on April 12, 2023, after two cycles of this combination therapy demonstrated a significant partial response (PR), with a reduction in the sum of target lesion diameters by 68.5% (**Table 1**). The pancreatic lesion exhibited marked shrinkage with a notable shift from a mixed solid-cystic to a predominantly solid composition, alongside continued regression of the hepatic and nodal metastases (**Figure 1d**).

**Figure 3:**
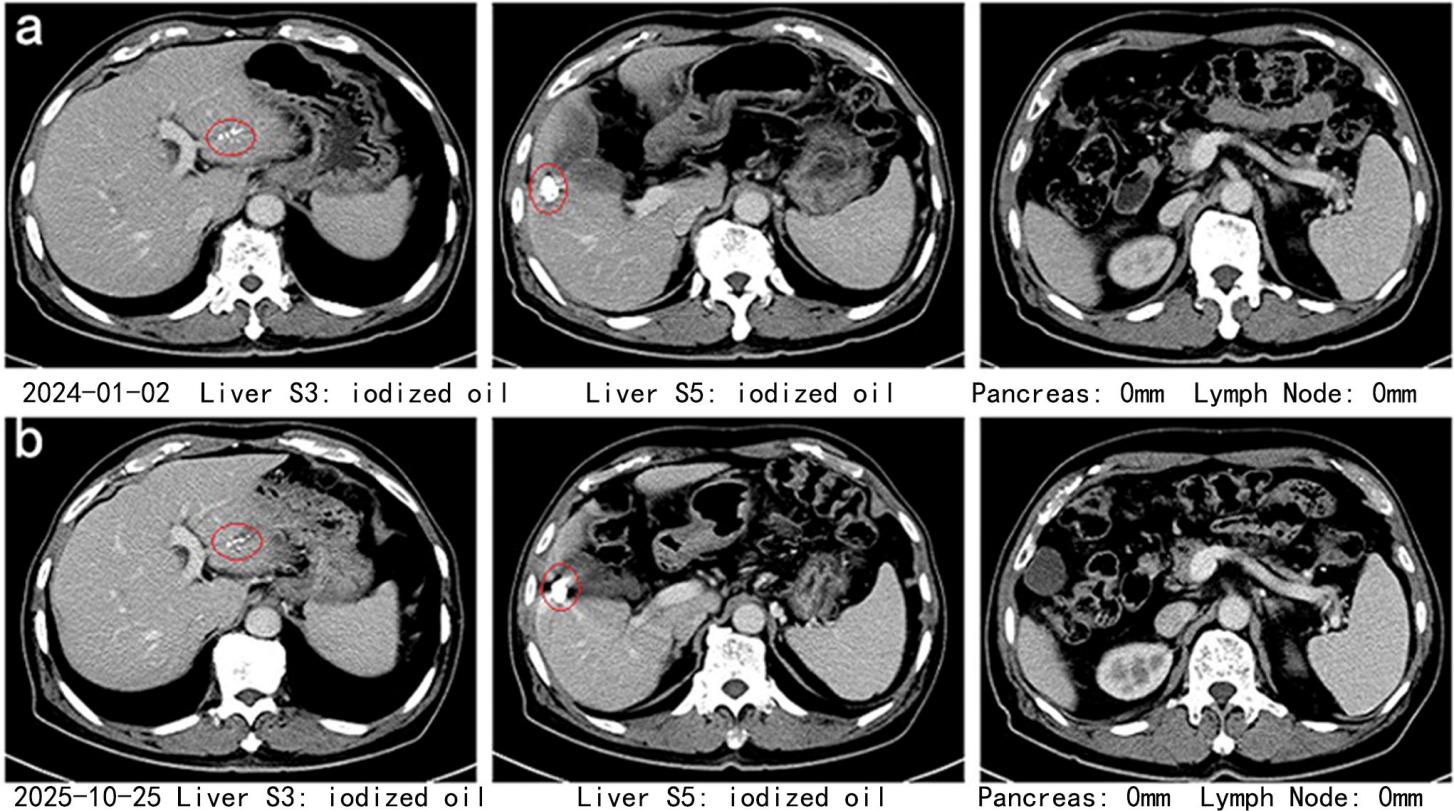
Long-term follow-up imaging confirming sustained complete response. **(a)** CT scan (January 2024) and **(b)** CT scan (October 2025) demonstrated a sustained complete response (CR), with complete resolution of the previously seen pancreatic primary lesion and metastatic lymph nodes. Only stable, non-enhancing iodized oil deposits remain in the liver, confirming the absence of viable tumor (**Table 1**).

Due to financial constraints, cadonilimab was discontinued. The patient subsequently received two cycles of nab-paclitaxel and oxaliplatin alone, which were complicated by grade III myelosuppression (assessed by the Common Terminology Criteria for Adverse Events [CTCAE] version 5.0), leading to the cessation of all anti-tumor therapy. Remarkably, surveillance CT on June 29, 2023, showed a profound reduction in the enhancing tumor burden (sum of diameters decreased by 89.9% from baseline), assessed as a near-complete response (near-CR) (**Table 1**, **Figure 1e**), with only residual non-enhancing changes (**Table 3**). Remarkably, follow-up imaging on January 3, 2024, confirmed the complete disappearance of all enhancing lesions, establishing a confirmed complete response (CR) (**Figure 3a**). This CR has been maintained for over 24 months, with the most recent scan in October 2025 showing no evidence of recurrence (**Tables 1**, **2**, **Figure 3b**).

**Table 3:** The tumor markers’ levels.

Parameters	CA19-9 (0.00-39.00)U/ml	CEA (0.00-5.10)ng/ml	AFP (0.00-10.0)ng/ml	CA72-4 (0.00-6.90)U/ml	CA125 (0.00-35.00)U/ml
2022.10.27	11.15	3.26	168.80	1.17	12.83
2023.01.05	13.17	5.01	81.11	1.08	8.52
2023.02.06	13.40	3.26	582.00	1.67	30.20
2023.02.27	13.90	6.51	481.00	1.79	15.10
2023.03.21	16.80	5.77	246.00	3.35	10.20
2023.04.11	12.60	5.15	18.90	6.60	16.30
2023.06.28	10.90	5.51	3.54	7.23	15.20
2024.01.02	15.50	3.81	2.10	6.68	11.13
2024.10.20	12.30	4.44	2.30	7.16	13.60

## Discussion

This report presents, to our knowledge, the first case of metastatic MSS PCAC achieving a sustained CR with cadonilimab plus chemotherapy. This exceptional response in a tumor type notoriously refractory to ICIs challenges prevailing therapeutic paradigms and warrants mechanistic exploration.

The efficacy observed here likely stems from a synergistic multi-hit strategy targeting the immunosuppressive PCAC microenvironment. First, cadonilimab’s unique mechanism is critical. Its symmetrical tetravalent structure enables high-avidity, simultaneous blockade of both PD-1 and CTLA-4 (14). This dual targeting is particularly relevant in the MSS PCAC microenvironment, which undergoes a dynamic transformation from an early CD8+ T cell-inflamed phenotype to a late, immunosuppressive milieu dominated by regulatory T cells (Tregs) and myeloid-derived suppressor cells (MDSCs) (15). Advanced imaging and response monitoring techniques, such as transformer-based pathology analysis (16) and CEA-targeted nanobody PET (17), may further improve the assessment of treatment response to bispecific antibodies in MSS solid tumors. Preclinical data suggest that by concurrently blocking these two pathways, cadonilimab not only reverses T-cell exhaustion but also attenuates Treg-mediated suppression, leading to synergistic T-cell activation (18). Furthermore, its Fc domain may mediate antibody-dependent cellular cytotoxicity (ADCC) via engagement of Fcγ receptors on innate immune cells (14), a mechanism particularly crucial for targeting PC tumors that frequently exhibit low PD-L1 expression (15). Macrophage repolarization from M2 to M1 phenotype has been shown to enhance bispecific antibody efficacy in ‘cold’ solid tumors (19). Preclinical studies demonstrate that bispecific antibody constructs exhibit superior efficacy compared to combinations of monoclonal antibodies in reversing the expression of T cell exhaustion markers (e.g., TIM-3 and LAG-3) (14), promoting the differentiation of stem-like T cells into effector T cells, and sustaining the duration of anti-tumor immune responses (18). The safety profile of monoclonal antibody-based immunotherapy in AFP-high gastrointestinal tumors has also been well documented (20). Critically, cadonilimab has shown promising clinical efficacy specifically in MSS tumors (21–23), providing a strong rationale for its application in our MSS PCAC case.

Second, the contribution of chemotherapy as an immunosensitizer is likely essential. Gemcitabine and nab-paclitaxel are known to induce immunogenic cell death, releasing tumor antigens to prime T-cell responses in a tumor with low neoantigen burden (24). They can also selectively deplete immunosuppressive cells like MDSCs and Tregs, thereby mitigating a key barrier to ICI efficacy (18, 25). Moreover, gemcitabine has been shown to promote the polarization of M1-type macrophages, actively shifting the microenvironment toward an immunostimulatory state (18). The phase II data showing a 50% objective remission rate (ORR) with nivolumab plus gemcitabine/nab-paclitaxel further supports the synergistic potential of such combinations (25). Deep learning-based prediction of MSI/TMB status has been validated in digestive tract cancers, further supporting the reliability of NGS in confirming MSS status in ‘cold’ tumors such as MSS PCAC (26).

Third, the synergy may be rooted in the specific disruption of a key immunosuppressive metabolic-epigenetic circuit. A recent research by Yang, J. et al. (27) delineated a “lactate-H3K18la-ACAT2” feedback loop in pancreatic cancer, wherein glycolysis-derived lactate promotes histone H3K18 lactylation, activating the cholesterol metabolism enzyme ACAT2. This, in turn, stabilizes MTCH2 to suppress mitochondrial respiration, further amplifying lactate generation (27); simultaneously, it promotes M2 macrophage polarization via cholesterol-laden extracellular vesicles (EVs) (28). In our case, chemotherapy (e.g., gemcitabine) may inhibit lactic acid production, while cadonilimab-restored CD8+ T cells can secrete IFN-γ to downregulate ACAT2. This dual attack potentially breaks the vicious cycle, reversing immunosuppression. This model is further supported by evidence that chemotherapy may modulate immunosuppressive EV secretion, while revitalized T cells eliminate EV-high tumor cells, collectively ameliorating immune evasion (18, 28).

In addition, several atypical clinical and pathological features in this case warrant discussion.

Differential Diagnostic Considerations. An important differential diagnosis in this case is acinar cell carcinoma (ACC), given the tumor’s acinar morphological features and the known increased immunogenicity of ACC which might confound the interpretation of immunotherapy response. However, several lines of evidence definitively excluded ACC. First, morphologically, the tumor lacked the characteristic solid, cellular architecture and pure acinar patterns typical of ACC, instead showing a predominant multicystic and glandular architecture. Furthermore, the tumor’s multicystic architecture, evident on imaging and confirmed pathologically, is a characteristic feature of pancreatic cystadenocarcinoma and further supports this diagnosis. Second and definitively, the tumor cells were immunohistochemically negative for the acinar lineage markers trypsin. Third, the overall immunophenotype (CK7+/CK20- with patchy CDX2) and the clinical-radiological presentation as a cystic neoplasm were consistent with PCAC. Therefore, the integrated diagnosis of PCAC is robust. Consequently, the observed profound and durable response to cadonilimab plus chemotherapy pertains to a classic MSS/pMMR, PD-L1-negative pancreatic cystadenocarcinoma, underscoring the potential efficacy of this therapeutic strategy even in a pancreatic cancer subtype historically considered resistant to immune checkpoint inhibition.

Notably, a heterogeneity in the proliferative index was observed, with the Ki-67 being higher in the liver metastasis (60%) compared to the primary tumor (30%). The Ki-67 heterogeneity suggests that metastatic clones may have acquired higher proliferative capacity, possibly through chemo-selection or intrinsic tumor evolution. This aggressive phenotype in the metastatic site may have contributed to the differential response to TACE versus systemic therapy, but ultimately responded favorably to cadonilimab-based combination.

The elevated AFP, uncommon in PCAC, suggests a hepatoid differentiation, which may have contributed to the distinct treatment response profile. Although the GALAD score is a validated biomarker for hepatocellular carcinoma, its role in AFP-producing pancreatobiliary tumors remains unestablished and was therefore not applied in this case. This morphological uniqueness is further supported by the cystic nature of both primary and metastatic lesions, potentially explaining the differential response to TACE versus initial systemic chemotherapy. While TACE contributed to the initial control of liver metastases, the subsequent near-complete and complete remissions were attributable to the second-line cadonilimab-based immunotherapy combined with chemotherapy, and this sequential contribution does not reflect any procedural bias. Finally, initial IHC findings suggestive of Lynch syndrome were conclusively ruled out by NGS, confirming MSS status and excluding a hereditary basis. Moreover, awareness of immune-related adverse events such as cholangitis is essential when applying ICIs in gastrointestinal malignancies (29).

Notably, the key predictive biomarkers—MSS/pMMR and PD-L1-negative status—were entirely concordant between primary and metastatic sites. This uniformity confirms that the exceptional response to cadonilimab occurred in a patient with a systemically MSS tumor burden, which is critical for interpreting the clinical relevance of our findings. Although minor immunohistochemical heterogeneity was observed (e.g., focal CDX2 expression in the metastasis), a phenomenon recognized as part of pancreatic cancer’s phenotypic plasticity potentially driven by epigenetic modifications or microenvironmental selection (30, 31), such variation does not undermine the diagnostic consistency established by core markers (CK7+/CK20-) and the negative pancreatic enzyme markers or the predictive concordance of MSS and PD-L1 status. Thus, the central conclusion remains strongly supported: the profound and sustained response in this case underscores the potential efficacy of cadonilimab-based therapy in this challenging patient population.

A limitation of this study is the lack of pre- and post-treatment tumor biopsies for immune correlative analyses, including TMB, neoantigen load, and TIL quantification, due to financial and ethical constraints. Should disease progression occur in the future, we plan to perform repeat biopsy to explore the immunological mechanisms underlying the ‘cold-to-hot’ transition. The lack of pre- and post-treatment gut microbiome (stool NGS) and EV data is a limitation. Emerging evidence suggests that gut microbiota composition may influence ICI response, and EV-mediated crosstalk can remodel the TME (32). Future prospective studies incorporating these analyses are warranted to better understand the mechanisms of response in MSS PCAC.

While this single case demonstrates a remarkable and durable complete remission, the findings should be interpreted with caution. Generalizability is limited by the n=1 design and the rarity of PCAC. Multi-center clinical trials are urgently needed to validate the efficacy of cadonilimab-based regimens in MSS PCAC and to identify predictive biomarkers.

In summary, this case provides pioneering evidence that cadonilimab, synergizing with chemotherapy, can induce profound remission in MSS PCAC. The efficacy stems from a multi-faceted synergy: the bispecific antibody restores T-cell function and mediates ADCC, while chemotherapy liberates antigens and reprograms the immunosuppressive microenvironment, together disrupting a key metabolic-epigenetic circuit. This clinical success warrants validation in prospective trials and prompts investigation into optimal treatment sequencing, novel metabolic targets, and the role of microbiota and extracellular vesicles in shaping the response to such combination strategies.

## Data Availability

The original contributions presented in the study are included in the article/supplementary material. Further inquiries can be directed to the corresponding author.
