# Correction: Converting an immunologically “cold” tumor: Exceptional response to cadonilimab plus chemotherapy in microsatellite-stable pancreatic cystadenocarcinoma

**DOI:** 10.3389/fimmu.2026.1897164

**Published:** 2026-06-10

**Authors:** Chunxiao Ni, Jiaju Xu, Yu Pang, Jiaju Xu

**Affiliations:** 1 Department of Minimally Invasive Oncology, Tai’an City Central Hospital, Tai’an, Shandong, China; 2 Department of Pediatrics, Yantai Yuhuangding Hospital, Yantai, Shandong, China; 3 Department of Pathology, Tai’an City Central Hospital, Tai’an, Shandong, China; 4 Department of Medical Oncology, Tai’an City Central Hospital, Tai’an, Shandong, China

**Keywords:** cadonilimab, bispecific antibody, pancreatic cystadenocarcinoma, microsatellite stable, complete remission, immunotherapy

Author "Jiaju Xu (second author)" was erroneously omitted as equal contributing author. The original version of this article has been updated.

